# The AF-2 cofactor binding region is key for the selective SUMOylation of estrogen receptor alpha by antiestrogens

**DOI:** 10.1016/j.jbc.2022.102757

**Published:** 2022-11-30

**Authors:** Amandine Vallet, Mohamed El Ezzy, Marine Diennet, Salwa Haidar, Michel Bouvier, Sylvie Mader

**Affiliations:** 1 Institut de Recherche en Immunologie et Cancérologie (IRIC), Université de Montréal, Montréal, Québec, Canada; 2 Département de Biochimie et Biologie Moléculaire, Université de Montréal, Montréal, Québec, Canada

**Keywords:** breast cancer, estrogen receptors, selective estrogen receptor downregulators (SERDs), SUMOylation, protein inhibitor of activated STAT 1 (PIAS1), AEs, Antiestrogens, AF-2, Activation Function-2, AZD, AZD9496, Baz, Bazedoxifene, BRET, Bioluminescence Resonance Energy Transfer, DMEM, Dulbecco's modified Eagle's Medium, ECL, Enhanced Chemiluminescence, E2, 17β-Estradiol, ERα, Estrogen Receptor α, ERβ, Estrogen Receptor β, EREs, Estrogen Responsive Elements, F, Fulvestrant, FBS, Fetal Bovine Serum, FBS-T, Treated Fetal Bovine Serum, GDC, GDC-0927, H3/4/12, helices 3/4/12, HEK293, Human Embryonic Kidney, Las, Lasofoxifene, LBD, Ligand-Binding Domain, Lys, Lysine, OHT, 4-hydroxytamoxifen, PEI, Polyethylenimine, PIAS1, Protein Inhibitor of Activated STAT 1, Ral, Raloxifene, RLucII, Renilla Luciferase II, RU, RU58,668, SENP1, SENtrin-specific Protease 1, SERMs, Selective Estrogen Receptor Modulators, SERDs, Selective Estrogen Receptor Downregulators, SUMO, Small Ubiquitin-related MOdifier, Tam, Tamoxifen, wt, wildtype, YFP, Yellow Fluorescent Protein

## Abstract

Antiestrogens (AEs) are used to treat all stages of estrogen receptor (ER)-positive breast cancer. Selective estrogen receptor modulators such as tamoxifen have tissue-specific partial agonist activity, while selective estrogen receptor downregulators such as fulvestrant (ICI182,780) display a more complete antiestrogenic profile. We have previously observed that fulvestrant-induced ERα SUMOylation contributes to transcriptional suppression, but whether this effect is seen with other AEs and is specific to ERα is unclear. Here we show that several AEs induce SUMOylation of ERα, but not ERβ, at different levels. Swapping domains between ERα and ERβ indicates that the ERα identity of the ligand-binding domain helices 3 and 4 (H3-H4 region), which contribute to the static part of the activation function-2 (AF-2) cofactor binding groove, is sufficient to confer fulvestrant-induced SUMOylation to ERβ. This region does not contain lysine residues unique to ERα, suggesting that ERα-specific residues in H3-H4 determine the capacity of the AE-bound ERα ligand-binding domain to recruit the SUMOylation machinery. We also show that the SUMO E3 ligase protein inhibitor of activated STAT 1 increases SUMOylation of ERα and of ERβ containing the H3-H4 region of ERα, but not of ERβ. Together, these results shed new light on the molecular basis for the differential capacity of selective estrogen receptor modulators and selective estrogen receptor downregulators to suppress transcription by ERα.

Two thirds of breast tumors express estrogen receptor α (ERα), a hormone-activated transcription factor that mediates the oncogenic effects of estrogens in these tumors, including the upregulation of proliferative genes. Estrogen receptor β (ERβ) shares conserved sequences and functional properties with ERα but is poorly expressed in most breast tumors and was reported to oppose the proliferative effects of estrogens when overexpressed in these cells (1, 2, 3, 4).

Upon binding to agonists such as 17β-estradiol (E2), ERs undergo a conformational change that induces binding as dimers to estrogen responsive elements (EREs) in the regulatory regions of target genes, resulting in recruitment of coactivator complexes that mediate histone modifications, chromatin remodeling, and recruitment of the basal transcription machinery (5, 6). Crystal structures of the ligand-binding domain (LBD) of ERs with different ligands revealed conserved helices whose folding generates the ligand-binding pocket. E2 binding stabilizes folding of the C-terminal helix 12 (H12) back onto the ligand binding cavity. The positioning of helix 12 above the ligand binding cavity exposes a set of amino acids essential for coactivator binding, which includes residues of helices 3, 4, 5 [the static part of activation function-2 (AF-2)] as well as from H12 (the mobile part of AF-2). Coactivators recruited by ERα AF-2 include the histone acetyl transferase complex p160-CBP/p300-pCAF, the histone remodeling complex SWI/SNF, and the mediator complex (5, 6).

Antiestrogens (AEs) are competitive ER inhibitors that prevent transactivation and block estrogen-dependent tumor growth. AEs are usually classified into two groups, SERMs (selective estrogen receptor modulators) and SERDs (selective estrogen receptor downregulators), although AEs appear to present a spectrum of partial agonist and receptor downregulating properties (7). SERMs, including tamoxifen (Tam), the first AE used successfully for breast cancer treatment, inhibit activity of ERs in a tissue and gene-specific manner, exerting partial agonist effects on ER-mediated transcription. Tam is used in the clinic for the treatment of all stages of primary breast cancer and is approved for the prevention of breast cancer in women with high risk for the disease (8, 9). Tam acts as a partial ER agonist in the uterus and bone in animal models and is associated with an increased risk of endometrial cancer in breast cancer patients (10, 11, 12, 13, 14, 15, 16). These properties have been associated with transcriptional suppression of the AF-2 activation function, located in the LBD, while the N-terminal activation function 1 is thought to remain active in a gene-specific and cell-specific manner, leading to partial agonist activity of Tam and other SERMs in bone, in endometrial cells, and in breast cancer cells (16, 17, 18, 19, 20, 21, 22). Indeed, the SERM side chain prevents H12 from acting as a lid to the ligand binding cavity. Instead H12 is repositioned to the coactivator binding groove (23, 24). This drastic conformational change prevents the binding of coactivators by AF-2 and favors the recruitment of corepressors SMRT and NCoR (25, 26, 27, 28, 29).

Fulvestrant (ICI 182,780) is a 7-alkylsulfinyl analog of E2 initially described as a pure AE that does not exhibit partial agonist activity in model systems and can remain active in cell lines selected for resistance to Tam (8, 30, 31, 32, 33), suggesting that it has distinctive properties not shared by Tam. Fulvestrant is approved in the clinic under the name Faslodex in the metastatic setting or as a second-line therapeutic option for endocrine therapy–resistant patients (34). The crystal structure of the rat ERβ receptor bound to a fulvestrant analog, ICI164,384, revealed interaction of its long side chain with the coactivator binding groove, the conformation of H12 being undefined (35). Whether this structural feature is conserved in human ERα is unclear, as no structure of fulvestrant or analogs with long side chains in complex with ERα has been reported. Its relevance to the mechanism of action of fulvestrant as a SERD is also uncertain, as fulvestrant was reported not to induce ERβ degradation (36). Nevertheless, use of a series of ICI164,384 derivatives with variable side chain lengths revealed that the capacity of the side chain to reach into the coactivator binding groove was associated with pure antiestrogenicity in a reporter assay in hepatic HepG2 cells (37).

The superior transcriptional suppression observed in the presence of fulvestrant has been explained by its capacity to induce ERα ubiquitination and accelerated proteasomal turnover of ERα in estrogen target tissues and in a variety of cell lines, whereas 4-hydroxytamoxifen (OHT, the active metabolite of Tam) stabilizes the ERα protein (31, 38, 39, 40, 41, 42). However, fulvestrant fully suppresses ERα transcriptional activity in transfected hepatic HepG2 cells without enhancing its degradation (43). We previously reported that fulvestrant also induces modification of ERα by small ubiquitin-related modifiers 1/2/3 (SUMO1/2/3) in ER+ breast cancer cells and identified four SUMO-modified sites in human ERα by mass spectrometry (37). Furthermore, induction of SUMOylation correlated with pure antiestrogenicity in our series of ICI164,384 derivatives with variable side chain lengths. Finally, preventing SUMOylation by overexpression of the SENtrin-specific Protease 1 (SENP1) deSUMOylase abolished the difference in transcriptional repression by fulvestrant and OHT in transfected ER-negative cells and in endogenously ER-expressing breast cancer cells (37, 44).

Here we set out to investigate whether fulvestrant and other AEs can induce SUMOylation of ERβ as well as ERα. Using specific ER–SUMO interaction assays based on bioluminescence resonance energy transfer (BRET) (37, 45), we show that several AEs can induce SUMOylation of ERα by SUMO1 and SUMO3 in live cells. Fulvestrant was the most efficacious AE tested for modification of ERα but was inactive with ERβ. We then explored the molecular basis for the observed receptor specificity of fulvestrant using ERα/ERβ chimeras. Our results highlight the key role of ERα residues lining the static part of the cofactor binding groove in enabling recruitment of the SUMOylation machinery, including members of the SUMO E3 ligase protein inhibitor of activated STAT (PIAS) family, to ERα in the presence of fulvestrant.

## Results

### Estrogen receptors are differentially modified in the presence of fulvestrant

**Figure 1:**
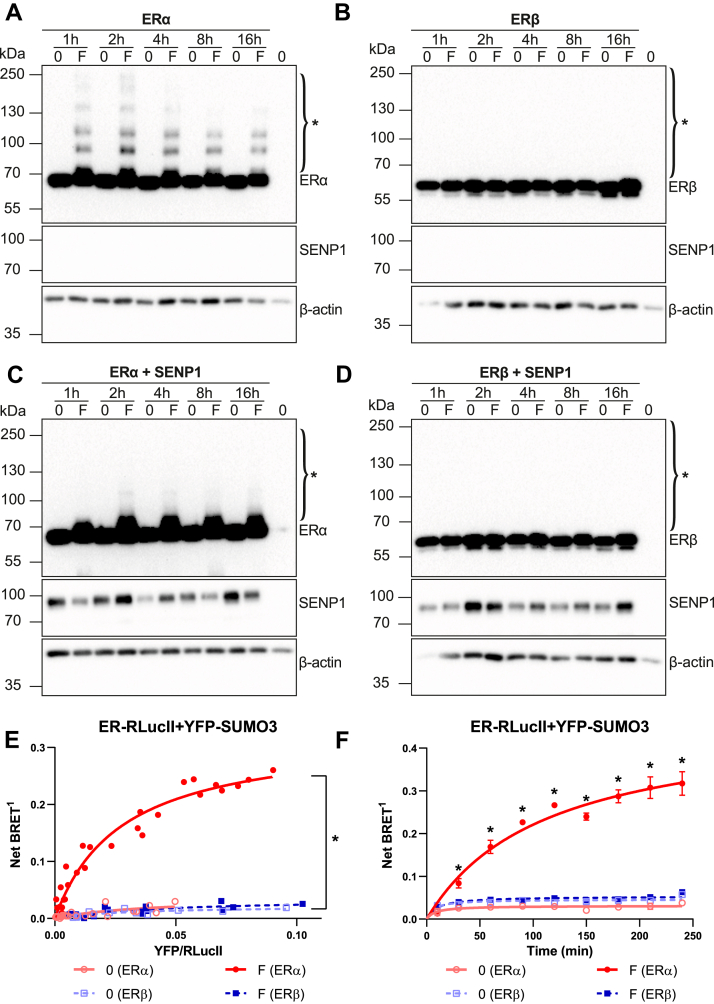
**Fulvestrant induces SUMOylation of ERα but not ERβ**. *A*–*D*, Western blot analysis of ERα and ERβ modifications after transient transfection of HEK293 cells with their respective expression vectors, with or without a SENP1 expression vector, and treatment of cells at different time points with fulvestrant (1 μM, F) or vehicle (0). ∗ indicates modified ERα forms. Blots are representative of three independent experiments. *E*, BRET titration curves performed by transient transfection of HEK293 cells with a fixed amount (50 ng) of ERα-RLucII or ERβ-RLucII and varying amounts of YFP-SUMO3 (0–800 ng) expression vectors, either in the presence of fulvestrant (1 μM, *F*) or vehicle (0) for 2 h. The net BRET^1^ ratio measured in live cells after addition of coelenterazine H (5 μM) is plotted as a function of measured YFP/RLuc ratios. The graph is a compilation of three independent experiments each performed in quadruplicates. Statistical analyses were performed using nonlinear regression analysis and comparison of Bmax values using one-way ANOVA and Tukey’s multiple comparison test in GraphPad Prism 6.07 (∗*p* < 0.05). *F*, BRET kinetics performed by transfecting a fixed amount of ERα-RLucII or ERβ-RLucII (50 ng) and YFP-SUMO3 (500 ng) expression vectors in HEK293 cells in the presence of fulvestrant (1 μM, *F*) or vehicle (0). Net BRET^1^ ratios were read at different time points (10–240 min) in live cells after addition of coelenterazine H (5 μM). Compiled results from three independent experiments (mean values ± SEM) are shown. Statistical analyses were performed using a Holm–Sidak’s multiple *t* test assuming the same scatter in GraphPad Prism 6.07 (∗*p* < 0.05). BRET, bioluminescence resonance energy transfer; ERα, estrogen receptor α; ERβ, estrogen receptor β; RLucII, Renilla luciferase II; SENP1, SENtrin-specific protease 1; SUMO, small ubiquitin-related MOdifier; YFP, yellow fluorescent protein.

To assess the impact of fulvestrant on modifications of ERα and ERβ, we first monitored their migration in Western analyses in transiently transfected human embryonic kidney 293 (HEK293) cells, which do not express detectable levels of ERα or ERβ. Treatment of cells transiently expressing ERα with fulvestrant (F) for 1, 2, 4, 8, or 16 h revealed a ladder of ER forms at higher molecular weight than the expected ∼65 kDa unmodified form (Fig. 1*A*). On the other hand, no such higher molecular weight bands were observed upon fulvestrant treatment of cells transfected with an ERβ expression vector at the same time points (Fig. 1*B*). To verify whether the modified bands correspond to SUMOylation of ERα, as previously reported (37), we co-transfected the deSUMOylase SENP1 together with expression vectors for either ERα or ERβ. Expression of SENP1 prevented the generation of higher molecular weight bands in the presence of fulvestrant (Fig. 1*C*). Co-expression of SENP1 did not modify the ERβ migration profiles in the presence or absence of fulvestrant (Fig. 1*D*). Neither ERα nor ERβ were downregulated under the conditions of the assay, whether in the absence or presence of SENP1.

To measure SUMOylation of ERα and ERβ in a semiquantitative manner in the presence of SERDs, we used our previously described BRET assays for interaction between ERα and SUMO1 or SUMO3 (37) and developed similar assays for interaction between ERβ and SUMO1 or SUMO3 in transiently transfected HEK293 cells. *Renilla* luciferase II (RLucII) was fused to the C terminus of either ERα or ERβ, and yellow fluorescent protein (YFP) was fused to the N terminus of either SUMO1 or SUMO3. Titration assays were performed by transiently transfecting a fixed amount of ERα/β-RLucII and increasing amounts of YFP-SUMO3 in HEK293 cells maintained in estrogen-depleted medium 2 days before transfection and for the duration of the assay. BRET ratios were quantified by measuring the ratio of light emitted by the fluorescent acceptor and the luciferase donor upon addition of the luciferase substrate coelenterazine H. Net BRET ratios correspond to the difference between the measured BRET ratios and those obtained from cells transfected with ERα/β-RLucII only. Net BRET ratios were plotted as a function of the measured ratios of total fluorescence/luminescence activities, reflecting expression of the fusion proteins. As previously reported (37), fulvestrant (F, 2 h) induced interaction of ERα-RLucII and YFP-SUMO3 in a saturable manner, while no significant signal was detected with control vehicle treatment (Fig. 1*E*; see Fig. S1*A* for statistically significant differences in BRETmax values calculated by nonlinear regression analysis for each saturation curve). On the other hand, no increased interaction was detected with ERβ-RLucII in the same experiment in the presence of fulvestrant *versus* its absence (Figs. 1*E* and S1*A*). Similar results were obtained using SUMO1 instead of SUMO3 (Fig. S2, *A* and *B*). In addition, use of a YFP-SUMO1G construct, in which YFP is fused to a SUMO1 mutant that cannot be conjugated (46), confirmed that the BRET signals observed with ERα result from covalent interaction (Fig. S3). Further, co-transfection of the SUMO E3 ligase protein inhibitor of activated STAT 1 (PIAS1) increased the BRET signal with SUMO1 but not SUMO1G (Fig. S3). Finally, similar results were obtained with an alternative ERα fusion construct in which ERα was fused C terminally to RLucII (RLucII-ERα), indicating that the observed SUMO BRET signal in the presence of fulvestrant is insensitive to the location of the RLucII moiety (Fig. S3).

We next used the SUMO BRET assay to characterize the kinetics of ERα modification by SUMO3 and determine whether ERβ modification is detected after longer incubation with pure AEs. Addition of fulvestrant resulted in a progressive increase in ERα interaction with SUMO3 over a 4 h treatment, but no significant increase in basal signal was detected with ERβ at any time point (Fig. 1*F*), consistent with the results observed by Western analysis (Fig. 1, *A* and *B*).

Together, these results indicate that ERα is specifically SUMOylated by SUMO1 and SUMO3 in the presence of fulvestrant.

### ERα SUMOylation is induced by AEs with long side chains or bulky hydrophobic terminal groups

**Figure 2:**
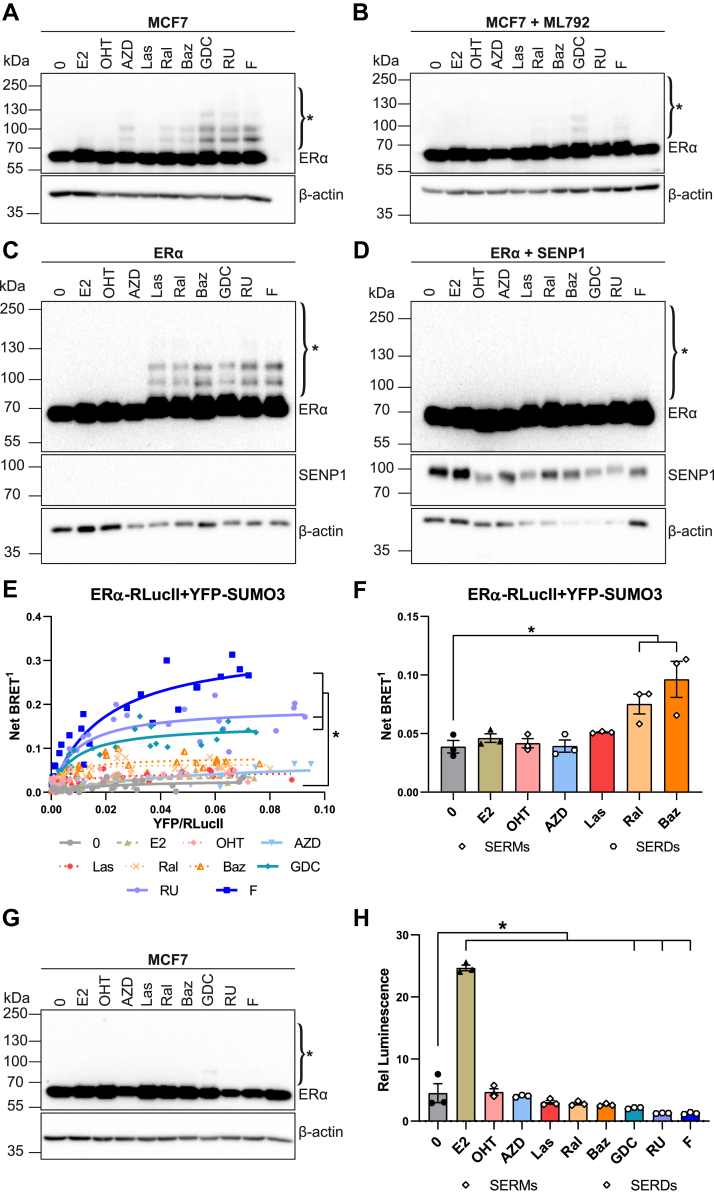
**Spectrum of ERα SUMOylation induced by antiestrogens**. *A* and *B*, Western blot analyses of endogenous ERα modifications after treatment with 17β-estradiol (E2, 25 nM) or with the acidic side chain SERD AZD9496 (AZD), the basic side chain SERMs 4-hydroxytamoxifen (OHT), lasofoxifene (Las), raloxifene (Ral), or bazedoxifene (Baz), the basic side chain SERD GDC-0927 (GDC), or the long side chain SERDs RU58,668 (RU) or fulvestrant (F) (1 μM), or with vehicle (0) for 1 h without (*A*) or with (*B*) pretreatment with the SAE inhibitor ML-792 (1 μM, 6 h). Blots are representative of three independent experiments. ∗ indicates modified ERα forms. *C* and *D*, western blot analyses of ERα modifications in transiently transfected HEK293 cells, in the presence or absence of SENP1. Cells were treated for 1 h with E2 (25 nM), antiestrogens (1 μM), or vehicle. Blots are representative of three independent experiments. ∗ indicates modified ERα forms. *E*, BRET titration curves performed by transient transfection of HEK293 cells with a fixed amount (50 ng) of ERα-RlucII and varying amounts of YFP-SUMO3 (0–800 ng) expression vectors, in the presence of E2 (25 nM), antiestrogens (1 μM), or vehicle for 2 h. The net BRET^1^ ratios measured in live cells after addition of coelenterazine H (5 μM) are plotted as a function of the YFP/RLuc calculated ratios. The curves are a compilation of three independent experiments, each point representing the mean values of technical quadruplicates. Bmax values derived from nonlinear regression analysis were calculated for each biological replicate, and statistical analysis was performed using one-way ANOVA and Tukey’s multiple comparison test in GraphPad Prism 6.07 (∗*p* < 0.05). *F*, BRET assays between ERα-RlucII and YFP-SUMO3 after treatment for 2 h with E2 (25 nM) or antiestrogens (1 μM) in HEK293 cells transfected with a constant amount of ERα-RlucII (50 ng) and YFP-SUMO3 (500 ng). The graph shows mean values ± SEM from three independent experiments performed in technical octuplicates. Statistical analyses were performed using a one-way ANOVA followed by Dunnett’s multiple comparison test in GraphPad Prism 6.07 (∗*p* < 0.05). *G*, Western blot analyses of endogenous ERα levels in MCF7 cells after treatment with 17β-estradiol (E2, 25 nM), antiestrogens (1 μM), or vehicle (0) for 6 h. Blots are representative of three independent experiments. ∗ indicates modified ERα forms. *H*, luciferase assays in HEK293 cells transiently cotransfected with an expression vector for ERα and a *GREB1*-ERE-Luc reporter vector. Cells were treated 24 h after transfection with vehicle, E2 (2.5 nM), or antiestrogens (1 μM) for another 24 h. The graph shows mean values ± SEM from three independent experiments performed in technical triplicates. Statistical analyses were performed using Holm–Sidak’s multiple *t* test assuming the same scatter in GraphPad Prism 6.07 (∗*p* < 0,05). BRET, bioluminescence resonance energy transfer; ERE, estrogen responsive element; ERα, estrogen receptor α; ERβ, estrogen receptor β; RlucII, Renilla luciferase II; SUMO, small ubiquitin-related modifier; SENP1, SENtrin-specific protease 1; SERDs, selective estrogen receptor downregulators; YFP, yellow fluorescent protein.

**Figure 3:**
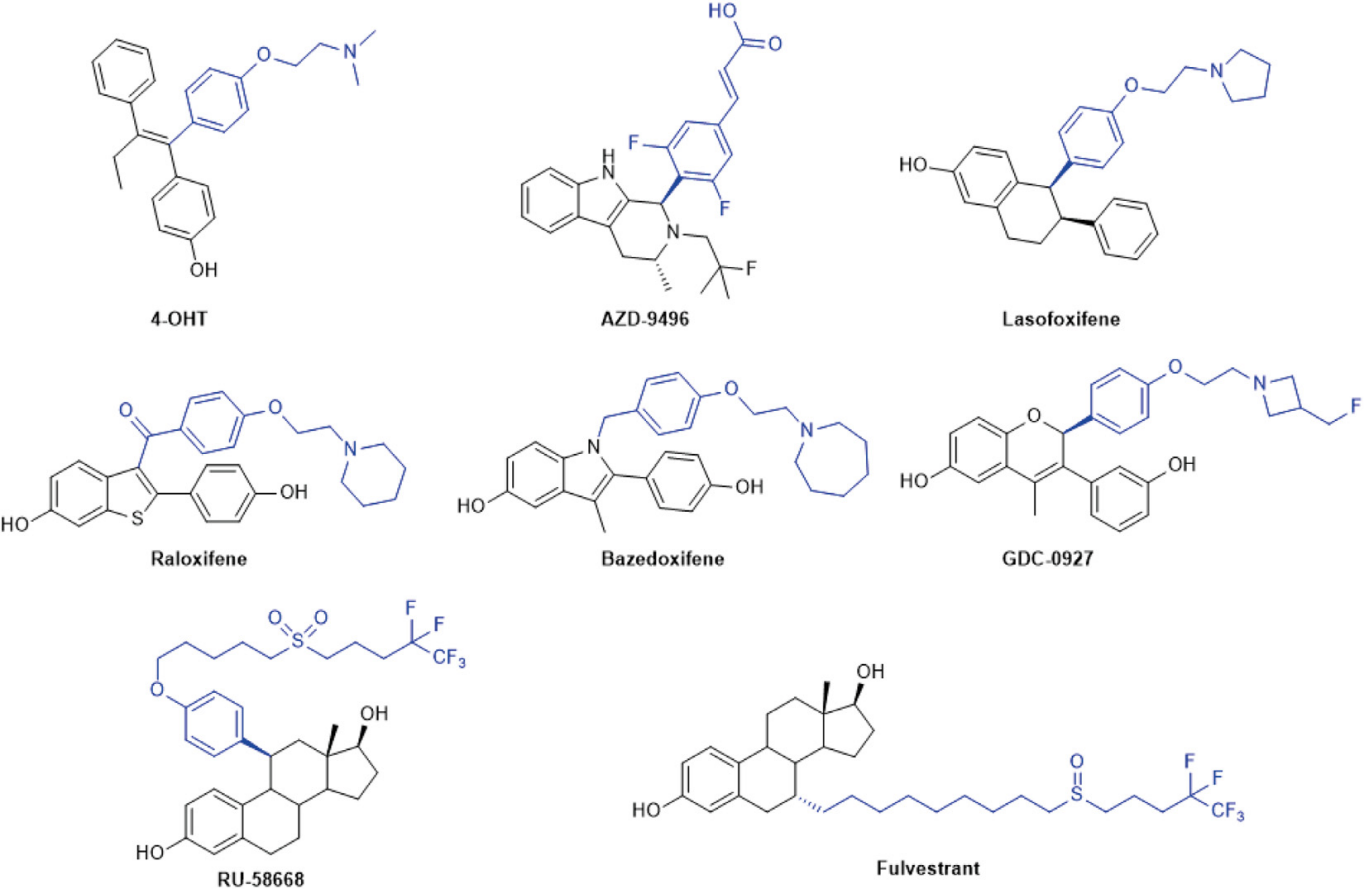
**Antiestrogens used in this study**. The side chain is highlighted in *blue*.

To investigate whether other AEs than fulvestrant can induce SUMOylation of ERα, MCF7 breast cancer cells, which express endogenous ERα, were treated with different steroid or steroid-like AEs for 1 h. As previously observed (37), OHT did not induce ERα modification (Fig. 2*A*). On the other hand, the SERM raloxifene (Ral) and the SERM/SERD bazedoxifene (Baz), which have bulkier side chain ends compared to OHT (Fig. 3; side chains highlighted in *blue*), induced low levels of ERα modifications. The SERD GDC-0927 (GDC) (Fig. 3), with reported SERD activity, as well as fulvestrant (F) and RU58668 (RU), a fulvestrant analog with the side chain linked to the steroid core at position 11β instead of 7α (Fig. 3) (7, 47, 48), efficiently induced ERα modifications (Fig. 2*A*). In contrast, the SERD AZD9496 (AZD), with a short side chain ending in a carboxylic acid (49) (Fig. 3), induced weaker but detectable modification levels (Fig. 2*A*). When cells were pretreated with an inhibitor of the unique SAE SUMO-activating enzyme (ML-792, 1 μM, 6 h), modifications were strongly reduced, indicating that they are dependent on an active SUMOylation system (Fig. 2*B*).

AE treatment of HEK293 cells transiently transfected with an ERα expression vector also resulted in the appearance of higher molecular forms with Ral, Baz, GDC, RU, and F. Differences with results observed in MCF7 included lack of detectable modifications by AZD, while lasofoxifene (Las) induced modifications (Fig. 2*C*). All higher molecular weight forms disappeared when the deSUMOylase SENP1 was cotransfected, similarly suggesting that these bands are SUMOylated forms (Fig. 2*D*). This was further confirmed using a BRET SUMO3 assay. Increasing BRET signals were detected with increasing acceptor/donor ratios (Fig. 2*E*). BRETmax values calculated by nonlinear regression analysis increased with Ral, Baz, GDC, RU, and F, statistical significance being reached for the last three treatments (N = 3, one-way ANOVA followed by a Tukey’s multiple comparison test; Fig. S1*B*). Using expression vector ratios in the saturation range, we repeated BRET assays with AEs OHT, AZD, Las, Ral, and Baz and observed significant increases in SUMOylation levels with Ral and Baz and a nonsignificant increase with Las compared to the absence of treatment (Fig. 2*F*). In all BRET assays experiments, AZD did not detectably induce the SUMO BRET signal, in agreement with the results from our Western analysis (Fig. 2, *C* and *F*). This suggests that low levels of SUMOylation observed with some AEs (AZD, Las) may be cell-context dependent, possibly due to the expression of different SUMO E3 ligases or deSUMOylases. Of note, in spite of its SERD activity in MCF7 breast cancer cells, AZD9496 has been reported to have estrogen-like uterotrophic activity in ovariectomized mice (50).

To investigate whether induction of SUMOylation correlates with induction of ERα degradation, a distinctive SERD property, we treated MCF7 cells with AEs for a longer duration (6 h), observing a drug-specific effect on ERα levels (Fig. 2*G*). Fulvestrant and RU reduced expression most drastically, followed by GDC-0927 and AZD9496. ERα levels in the presence of Ral and Baz were equal to those in the absence of treatment and slightly increased in the presence of OHT and Las (Fig. 2*G*). These results indicate that all compounds that induced degradation also induced SUMOylation, although the extent of degradation and SUMOylation do not correlate exactly at these time points. Of note, detection of modified bands is influenced by the amount of total receptor, complicating quantification.

As BRET assays offer a more quantitative evaluation of SUMOylation, we compared the impact of AEs on ERα SUMOylation and transcriptional activity using a reporter vector containing the proximal enhancer of the *GREB1* gene, an estrogen target in breast cancer cells (*GREB1*-ERE-Luc). Mirroring the differential regulation of this gene by OHT and fulvestrant in MCF7 cells (44), fulvestrant but not Tam displayed inverse agonist effect in this assay. Overall, the degree of transcriptional repression observed in this assay was proportional to the extent of SUMOylation detected in BRET assays (Figs. 2*H* and S1*B*).

Together, these results indicate that AEs can induce ERα SUMOylation in ER+ breast cancer cells and in transfected ER-negative cells with a range of efficacies correlating with the length of the AE side chain and/or the bulk of terminal hydrophobic groups, cell-specific effects being observed for some AEs at the lower end of the efficacy spectrum. Further, they suggest links between SUMOylation, induction of ER degradation, and a greater degree of transcriptional suppression.

### The E region of ERα is necessary for induction of SUMOylation of ERα/ERβ chimeras by fulvestrant

**Figure 4:**
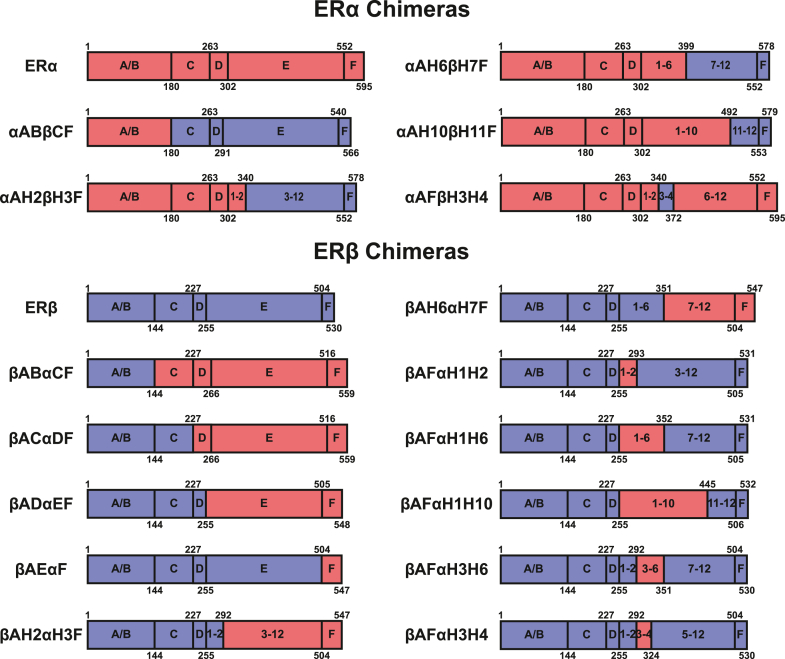
**ERα/ERβ chimera constructs**. Chimeras were constructed either fused to the N terminus of RLucII for BRET assays or unfused for Western analysis. BRET, bioluminescence resonance energy transfer; ERα, estrogen receptor α; ERβ, estrogen receptor β; RlucII, Renilla luciferase II.

**Figure 5:**
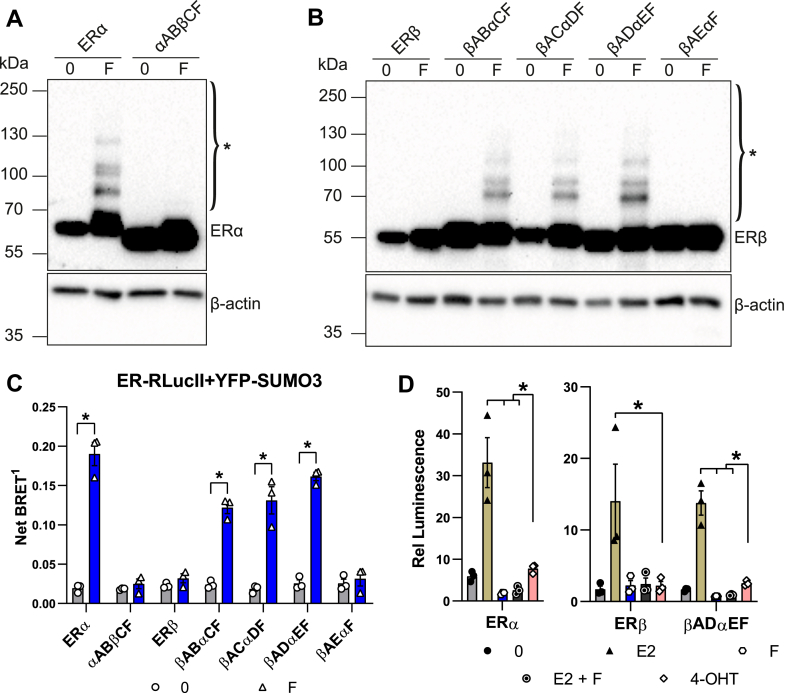
**The ligand-binding domain of ERα is required for induction of SUMOylation of chimeras by fulvestrant**. *A* and *B*, Western analyses of modification of ERα/β chimeras by fulvestrant. HEK293 cells were transiently transfected with expression vectors for ERα, ERβ, or chimeras and treated with fulvestrant (1 μM) or vehicle for 1 h. Blots are representative of three independent experiments. ∗ indicates modified ERα forms. *C*, SUMO3 BRET assay with ERα/β chimeras in HEK293 cells treated or not with fulvestrant (2 h, 1 μM). *D*, the graph shows mean values ± SEM from three independent experiments, each performed in triplicates. Luciferase assays in HEK293 cells transiently transfected with an expression vector for ERα, ERβ, or with the βADαEF chimera together with a *GREB1*-ERE-Luc reporter vector. Cells were treated 24 h after transfection with E2 (2.5 nM) with or without fulvestrant (1 μM; E2 + F), OHT, fulvestrant (1 μM each) or vehicle and assayed for luciferase activity after 24 h. The graph shows mean values ± SEM from three independent experiments performed in technical triplicates. Statistical analyses (*C* and *D*) were performed using Holm–Sidak’s multiple *t* test in GraphPad Prism 6.07 assuming the same scatter (∗*p* < 0,05). BRET, bioluminescence resonance energy transfer; ERα, estrogen receptor α; ERβ, estrogen receptor β; ERE, estrogen responsive element; OHT, 4-hydroxytamoxifen.

To map receptor domains implicated in the differential impact of AEs on ERα/ERβ modifications, we generated a set of chimeras exchanging different portions of the two receptors (Fig. 4). We first examined the potential importance of the A-B region, which has only 17% identity between the two receptors (51). However, a chimera containing the A-B region of ERα, but all other domains of ERβ (αABβCF, Fig. 4), was not modified in the presence of fulvestrant, as revealed by Western analysis (Fig. 5*A*) or BRET assays with SUMO3 (Fig. 5*C*) or SUMO1 (Fig. S4*A*). Conversely, the chimera containing the A-B region of ERβ fused to regions C-F of ERα (βABαCF, Fig. 4) was modified in both assays in the presence of fulvestrant (Figs. 5, *B*, *C* and S4*A*).

Increasing the ERβ content in the βABαCF chimera to include regions C and D yielded chimeras βACαEF and βADαEF (Fig. 4). Both chimeras were expressed to similar levels as the wildtype (wt) ERβ and βABαCF chimera and were modified, as detected in Western analysis (Fig. 5*B*) or in BRET assays with SUMO1/3 (Figs. 5*C* and S4*A*), indicating that the E-F region of ERα is sufficient for induction of modifications of ERα by fulvestrant. Further, the βAEαF (Fig. 4) chimera was not modified in Western analyses (Fig. 5*B*) or in SUMO BRET assays (Figs. 5*C* and S4*A*), indicating a requirement for the ERα LBD (region E). Titration curves using the βADαEF chimera confirmed saturable interaction with SUMO1, with a similar maximal signal intensity compared to wt ERα (Fig. S2, *A* and *B*).

Transient cotransfection of expression vectors for wt receptors or the βABαCF chimera in HEK293 cells together with the *GREB1*-ERE-Luc reporter vector indicated that consistent with previous reports (1, 2, 3), ERβ has lower basal and estrogen-induced activity compared to ERα (Fig. 5*D*). A chimeric receptor containing the AD region of ERβ but the EF-region of ERα had a similar pattern of basal and estrogen-induced activity (Fig. 5*D*). 4-OHT did not suppress basal activity with any construct. However, treatment with fulvestrant further inhibited activity compared to 4-OHT levels with wt ERα and the βADαΕF chimera, but not wt ERβ receptor (Fig. 5*D*).

Together, these results indicate that ERα-specific residues in the E region are necessary for induction of SUMOylation of ERα/ERβ chimeras by fulvestrant. This may reflect either the existence of receptor-specific SUMOylation sites in the ERα E region or the role of residues specific to ERα in this region in recruitment of the SUMOylation machinery.

### Amino acids specific to ERα in the H3-H4 region are necessary and sufficient for induction of ERβ SUMOylation by fulvestrant

**Figure 6:**
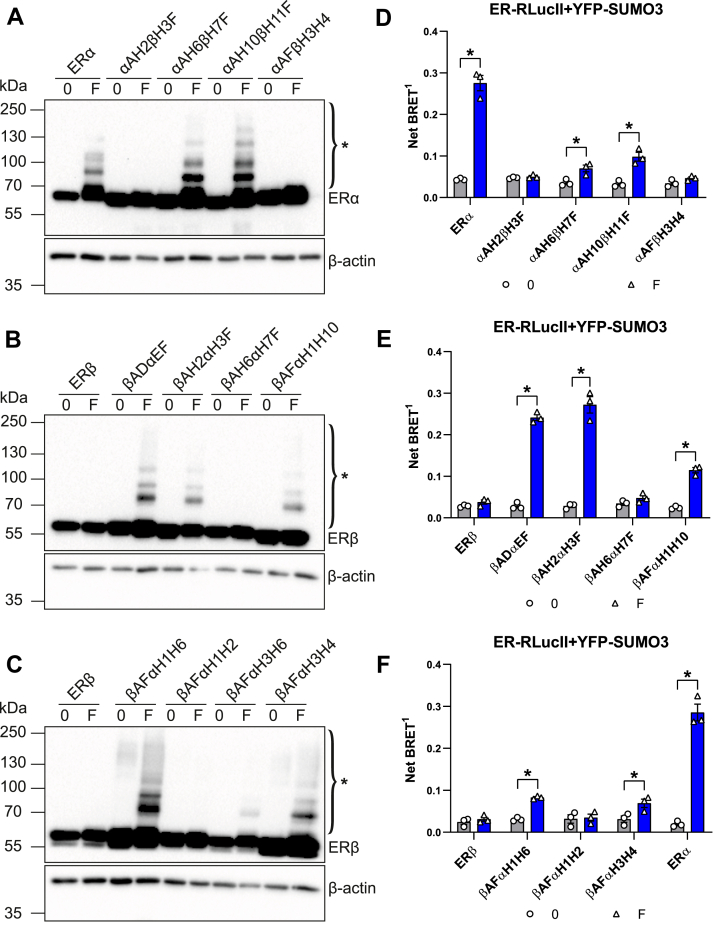
**The ERα identity of helices H3-H4 in the ligand binding domain is necessary and sufficient for induction of SUMOylation of chimeras by fulvestrant**. *A–C*, Western analyses of fulvestrant-induced modification of ERα/β chimeras. HEK293 cells were transiently transfected with expression vectors for ERα, ERβ, or chimeras as indicated and were treated with fulvestrant (1 μM) or vehicle for 1 h. Blots are representative of three independent experiments. ∗ indicates modified ERα forms. *D*–*F*, SUMO3 BRET assay for SUMOylation of ERα, ERβ, or chimeras in transfected HEK293 cells after 2 h of treatment with fulvestrant. The experiments were performed three times in technical triplicates. Graphs show mean values ± SEM from biological repeats. Statistical analyses were performed in GraphPad Prism 6.07 with a multiple *t* test using the Holm–Sidak method assuming the same scatter (∗*p* < 0,05). BRET, bioluminescence resonance energy transfer; ERα, estrogen receptor α; ERβ, estrogen receptor β; SUMO, small ubiquitin-related modifier.

To further map the regions of the ERα LBD contributing to induction of SUMOylation by fulvestrant, we created chimeras with fusion points within the LBD (Fig. 4). Induction of SUMOylation by fulvestrant was not observed when the fusion point between ERα and ERβ sequences was at the end of H2 (αAH2βH3F) but was restored when it was moved to the C-terminal side, including H6 (αAH6βH7F) or H10 (αAH10βH11F), in Western analyses (Fig. 6*A*), pointing to a requirement for ERα-specific sequences located in H3-H6. Further, replacing ERα sequences between H3-H4 by those of ERβ was sufficient to abolish induction of SUMOylation, further supporting that ERα-specific residues in this region are required for SUMOylation (Fig. 6*A*).

To map regions of ERα sufficient to confer SUMOylation of ERα by fulvestrant, we replaced ERα sequences by those of ERβ in the E-F region of the βADαΕF chimera, which is susceptible to SUMOylation in the presence of fulvestrant. Results indicate that region H3-F, but not H7-F of ERα (βAH2αΗ3F *versus* βAH6αΗ7F, Fig. 6*B*) was sufficient, indicating the importance of the H3-H6 region. Accordingly, helices H1-H10, H1-H6, H3-H6, and H3-H4, but not H1-H2, of ERα in an ERβ context led to induction of SUMOylation by fulvestrant (Fig. 6, *B* and *C*). These results indicate that ERα-specific residues within H3-H4 are sufficient for fulvestrant-induced SUMOylation.

Results obtained by Western analysis of modified ER forms were confirmed by BRET analysis of interaction between chimeras fusing ERα and ERβ within the LBD or chimeras derived from ERβ with swapped ERα sequences in the LBD and SUMO3 (Fig. 6, *D*–*F*) or SUMO1 (Fig. S4, *B*–*D*). Notably, significant SUMOylation of βAFαΗ1−Η10, βAFαΗ1−Η6, βAFαΗ3−Η4 (Fig. 6, *E* and *F*), but not αAFβΗ3−Η4 (Fig. 6*D*), was observed in the presence of fulvestrant.

Together, these results indicate that ERα-specific amino acids in the H3-H4 region, which together with helix H5 forms the static part of the cofactor binding groove, are both required and sufficient to confer induction of ERβ SUMOylation by fulvestrant.

### Role of the H3-H4 region and of PIAS proteins in the mechanisms of action of fulvestrant

**Figure 7:**
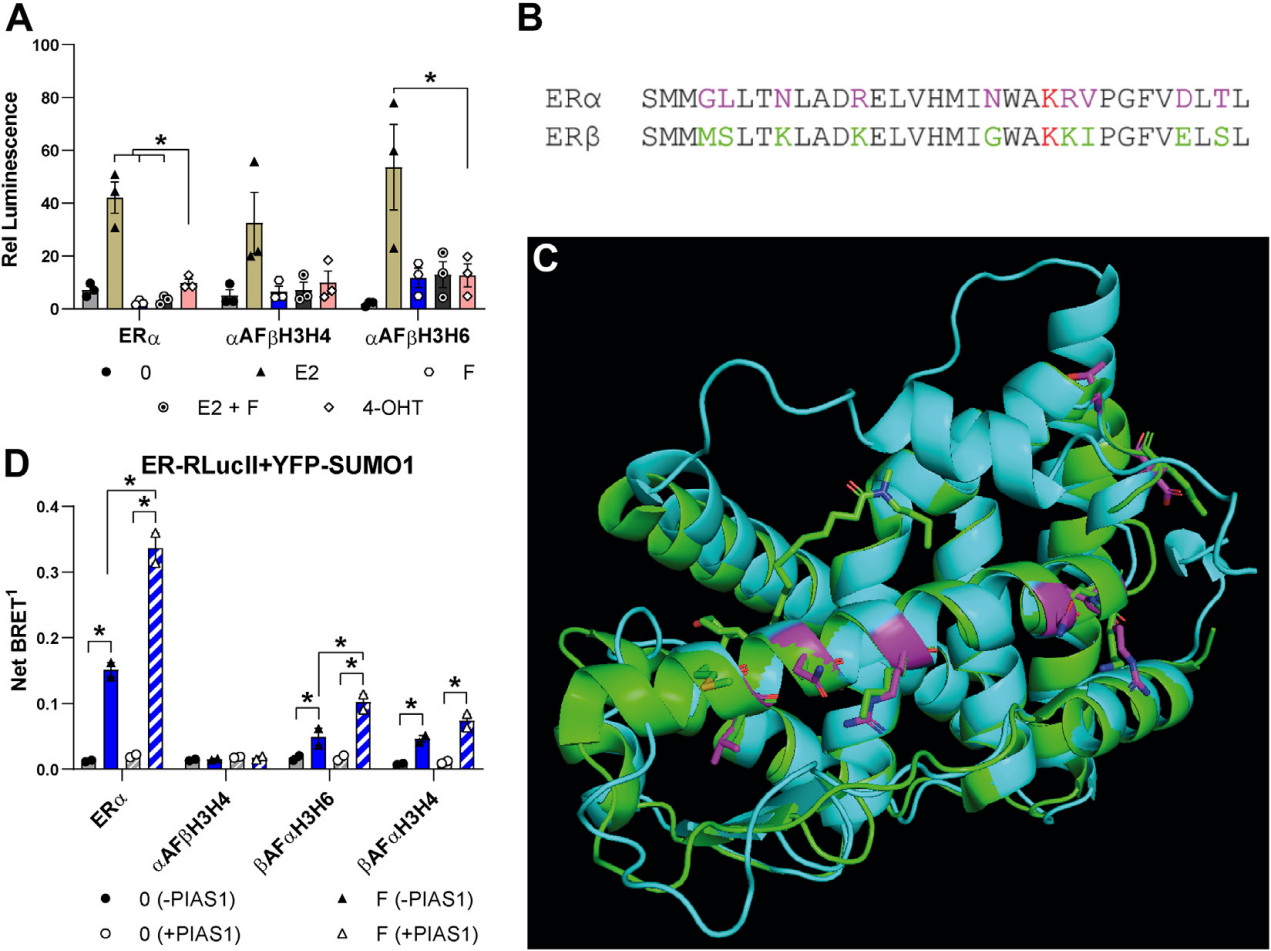
**Divergent amino acids in H3-H4 located on the outside rim of AF-2 confer induction of SUMOylation by PIAS1.***A*, luciferase assays in HEK293 cells transiently transfected with an expression vector for ERα, ERβ, αAFβH3H4, or αAFβH3H6 together with a *GREB1*-ERE-Luc reporter vector. Cells were treated 24 h after transfection with vehicle, E2 (2,5 nM) with or without fulvestrant (1 μM; E2 + F), OHT or fulvestrant (1 μM each) and assayed for luciferase activity after 24 h. The graph shows mean values ± SEM from three independent experiments performed in technical triplicates. Statistical analyses were performed using Holm–Sidak’s multiple *t* test in GraphPad Prism 6.07 assuming the same scatter (∗*p* < 0,05). *B*, residues in human ERα H3-H4 region diverging with rat ERβ are highlighted in magenta in the human ERα sequence and in *green* in the rat ERβ sequence. K362 in human ERα, conserved between the two receptors, is highlighted in *red*. *C*, superposition of the human ERα (from the 3ERT structure, *cyan*) and of the rat ERβ complexed to ICI164,384 (1HJ1 structure, *green*), whose side chain is inserted in the coactivator binding groove. Residues in the human ERα H3-H4 region diverging with rat ERβ are highlighted in *magenta* and those unique to rat ERβ are shown in *stick* representation (*green*). *D*, SUMO1 BRET was performed with ERα, ERβ, or chimeras exchanging the H3-H6 or H3-H4 regions in the presence (*striped bars*) or absence (*solid colors*) of PIAS1. The graph shows the mean values ± SEM from two independent experiments; each point represents the average from technical triplicates. Statistical analyses were performed using Holm-Sidak’s multiple *t* test assuming the same scatter in GraphPad Prism 6.07 (∗*p* < 0,05). BRET, bioluminescence resonance energy transfer; ERE, estrogen responsive element; ERα, estrogen receptor α; ERβ, estrogen receptor β; PIAS1, protein inhibitor of activated STAT 1; SUMO, small ubiquitin-related modifier.

We further investigated the role of the H3-H4 region in transcriptional activity of ERα in the presence of fulvestrant. The chimeras replacing the H3-H4 or H3-H6 region of ERα by the corresponding segment of ERβ (αAFβΗ3−Η4, αAFβΗ3−Η6) displayed a gain of transcriptional activity in the presence of fulvestrant, resulting in loss of the capacity of fulvestrant to repress transcriptional activity of ER more efficiently than OHT with the *GREB1*-ERE-Luc reporter construct in transiently transfected HEK293 cells (Fig. 7*A*).

The H3-H4 region (aa 340–372 of ERα) contains nine amino acids that diverge between human ERα and human or rat ERβ (Fig. 7*B*), located on the outer rim of the coactivator binding cleft in the static part of the AF-2 region (Fig. 7*C*). None of these changes include a lysine (Lys) residue specific to ERα, ERβ containing on the other hand three additional Lys residues compared to ERα. Of interest, several diverging amino acids in H3 are in close contact with the H2-H3 loop, whose positioning differs between human ERα and rat ERβ, which may affect the AF-2 stability. Alternatively, these residues could contribute to the specific recognition of AE-liganded ERα by interacting proteins recognizing changes induced within the cofactor binding groove by the ligand side chains. These interacting proteins could include components of the SUMOylation pathway involved in modification of ERα in the presence of AEs.

**Figure 8:**
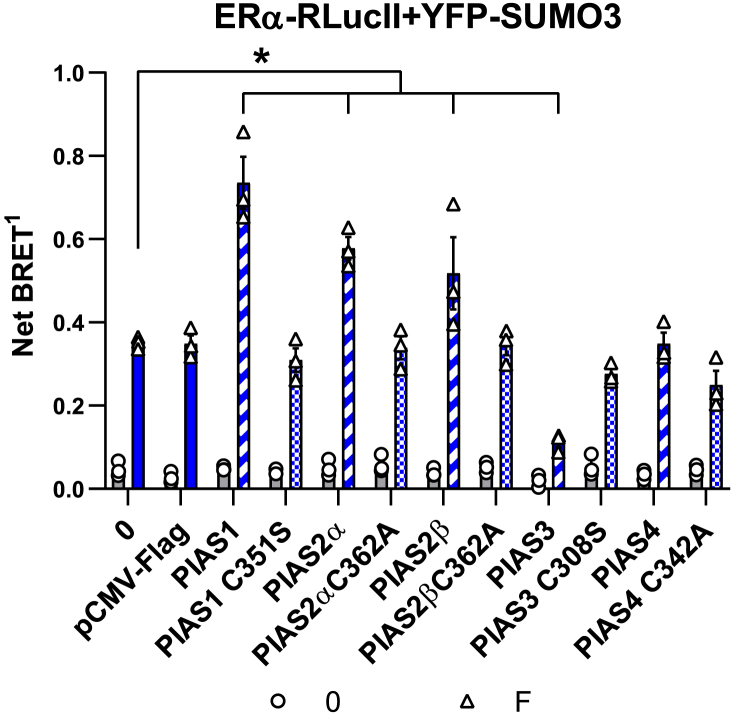
**PIAS proteins have a differential impact on SUMOylation of ERα in the presence of fulvestrant.** SUMO3 BRET assay for SUMOylation of ERα in the absence or presence of wt or catalytically dead mutant PIAS proteins in transfected HEK293 cells treated or not with fulvestrant for 2 h. HEK293 cells were transfected with a fixed amount of ERα (50 ng) and SUMO3 (500 ng) with or without overexpression (400 ng) of PIAS proteins (PIAS1, PIAS2α, PIAS2β, PIAS3, or PIAS4), catalytically dead mutants of PIAS proteins (PIAS1 C351S, PIAS2α or PIAS2β C362A, PIAS3 C308S or PIAS4 C342A) or empty vector (pCMV-Flag). The experiments were performed three times in technical triplicates. The graph shows mean values ± SEM from biological repeats. Statistical analyses were performed in GraphPad Prism 6.07 using a 2-way ANOVA with a Tukey’s multiple comparison test (∗*p* < 0,05). BRET, bioluminescence resonance energy transfer; ERα, estrogen receptor α; ERβ, estrogen receptor β; PIAS1, protein inhibitor of activated STAT 1; RlucII, Renilla luciferase II; SUMO, small ubiquitin-related modifier; YFP, yellow fluorescent protein.

As we observed that overexpression of PIAS1 increased ERα SUMOylation levels in response to fulvestrant (Fig. 7*D*), we tested whether PIAS1 also increased SUMOylation of the ERβ chimeras containing minimal ERα regions (Fig. 7*D*). Indeed, we observed significantly increased BRET signals for βAFαΗ3−Η6 and βAFαΗ3−Η4. On the other hand, no increase in BRET signal could be observed with the αAFβΗ3−Η4 chimera (Fig. 7*D*), demonstrating that the ERα H3-H4 region is necessary for recruitment of the SUMOylation machinery to estrogen receptors in the presence of fulvestrant. Moreover, we observed that, in addition to PIAS1, PIAS2α and PIAS2β also increased significantly the SUMOylation of ERα in a manner dependent on their SUMO E3 ligase activity (Fig. 8). On the other hand, PIAS3 repressed SUMOylation and PIAS4 did not have an impact.

These results suggest that the H3-H4 region is essential for recruitment of the SUMOylation machinery and that several PIAS proteins can induce SUMOylation of ERα in the presence of fulvestrant.

## Discussion

Here we report that the SERD fulvestrant has a different impact on SUMOylation of ERα and ERβ, as assessed in transfected HEK293 cells by Western analysis in the absence or presence of the deSUMOylase SENP1 and by BRET assays, which enable evaluation of ERα interaction with either SUMO1 or SUMO3. Interaction in BRET assays was abrogated by use of a nonconjugatable SUMO1 mutant and was increased by cotransfection with the SUMO E3 ligase PIAS1, demonstrating the dynamic range of the assay. Induction of SUMOylation was also observed to different degrees with other AEs with a spectrum of SERD activity, fulvestrant, RU58668, and new generation GDC-0927 being among the most efficacious AEs tested.

AEs display documented differences in pharmacological properties with ERα and ERβ. For instance, the SERM Tam has partial agonist activity with ERα but not ERβ, which has been attributed to the differential activity of their poorly conserved activation function 1 transcriptional activation regions (16, 17, 18, 19, 20, 21, 22). Conversely, fulvestrant induces degradation of ERα but not ERβ (36), although the mechanisms of this selectivity remain uncharacterized. Here we show that fulvestrant also induces SUMOylation of ERα but not ERβ and mapped residues key for this selectivity to the receptor LDBs.

The reported structures of the two receptor LDBs are similar in the presence of SERMs such as Ral, although H12 is more disordered in the ERβ structure (52). The structure of rat ERβ bound to a fulvestrant analog, ICI164,384, reveals a difference in LBD conformation compared to the SERMs OHT and Ral. The longer side chain of ICI164,384 protrudes from the ligand binding pocket and interacts with the coactivator binding groove, while this groove is occupied by H12 in Ral-bound rat ERβ as well as in OHT and Ral-bound human ERα (23, 24, 35, 52). H12 is not resolved in the ERβ structure with ICI164,384, suggesting mobility due to competition with the side chain for occupancy of the coactivator binding groove. There is currently no published structure of ERα bound to a fulvestrant analog. However, use of ICI164,384 analogs with different side chain lengths revealed that the capacity of the end of the chain to reach the coactivator binding groove was associated with complete suppression of transcriptional activity and induction of SUMOylation of ERα; analogs with shorter aliphatic side chain lengths, on the other hand, yielded partial agonist activity (37). Results obtained here with our series of AEs, indicating that the efficacy of induction of SUMOylation by AEs correlates with the presence of bulky or longer linear side chains, support this model. However, the observation of cell-specific SUMOylation induced by AZD9496, an AE with a short acidic side chain, suggests that alternative conformations can also induce SUMOylation. Of interest, a crystal structure obtained with another AE with a short side chain, GW5638, indicated formation of a capping hydrogen bond between the carbonyl of the AE acidic group and the peptidyl nitrogen of L536 at the N terminus of H12 (53). The resulting distortion in H12 positioning exposed hydrophobic amino acids at the N terminus of this helix to the solvent. Extended side chain reaching the coactivator binding groove may also interfere with positioning of the long hydrophobic residues that stabilize H12 in this groove.

Two alternative hypotheses may explain the different impact of fulvestrant on ERα and ERβ. The latter may lack critical SUMOylation residues, or differences in primary sequences between the two receptors may result in specific recruitment of the SUMOylation machinery by ERα. Of note, we previously mapped by mass spectrometry four ERα residues SUMOylated in the presence of fulvestrant, but their mutagenesis did not prevent SUMOylation, indicating the existence of multiple possible sites of SUMOylation (37). To identify structural determinants responsible for the different impact of fulvestrant on each receptor, we characterized chimeras between the two receptors for induction of SUMO modifications by fulvestrant. Regions A-B could be exchanged between the two receptors without affecting the specificity of fulvestrant-induced SUMOylation for ERα, consistent with our previous observation that A-B deletion in ERα does not affect SUMOylation in presence of fulvestrant (37). Region F, which is also poorly conserved between the two receptors (51), has been suggested to modulate the antagonist/agonist effectiveness of AEs by contributing to the partial activity of SERMs (54). However, its replacement in ERβ did not confer the capacity to be modified in the presence of SERD, and the presence of an ERβ F region in chimeras αAH6βH7F and αAH10βH11F indicates that the F region of ERα is not required for modifications to take place in the presence of fulvestrant. Rather, our results narrowed down the domain responsible for the different impact of fulvestrant on ERα and ERβ to sequences within region E, which corresponds to the LBD. Swapping of discrete E subregions between the two receptors demonstrated that replacement of H3-H4 in ERα by the corresponding region in ERβ yielded a chimera that did not display modification in Western or BRET analysis in the presence of fulvestrant. Conversely, introducing the ERα H3-H4 region in the context of ERβ was sufficient to confer to this receptor the capacity to be modified by fulvestrant. The lack of Lys residues specific to ERα *versus* ERβ in this region suggests a conformational role in enabling SUMOylation of ERα.

The increase in SUMOylation activity observed with several PIAS proteins, which are ubiquitously expressed proteins, suggests that SUMOylation of ERα is likely to take place in a variety of tissue types, but that its efficacy could be modulated as a function of relative PIAS protein levels, especially for weak inducers such as Las and AZD9496. Future characterization of the recruitment mechanisms of the SUMOylation machinery by ERα in the presence of AEs will clarify the contribution of receptor-specific sequences in SUMOylation, and the range of tissues in which these interactions take place. In addition, it will be of particular interest to investigate the potential links between induction of mono-, multi- or poly-SUMOylation and ubiquitination of ERα (55) by SERDs. Ligand-specific differences in SUMOylation patterns could result in different rates of ubiquitination and degradation. Mono-SUMOylation may result in competition between the two modification marks for the same residues. On the other hand, SUMO-targeted ubiquitin ligases such as RNF4 or RNF111 can target SUMOylated proteins for polyubiquitination and degradation. Cell-specific differences in expression levels of SUMO-targeted ubiquitin ligases such as RNF4 could therefore account for the reported cell specificity in ERα degradation by SERDs (43, 56).

The greater efficacy of SERDs *versus* SERMs at repressing transcription from ERα in breast cancer cells as well as in other tissues has been attributed to induction of ERα degradation, although this remains disputed as transcriptional repression can be observed in the absence of degradation (16, 19, 20, 21, 22). Our observation that SUMOylation levels with different AEs and with ERα/ERβ chimeras correlate with suppression of transcription mediated by an ERE-containing enhancer of *GREB1*, a natural estrogen target gene in estrogen responsive tissues, supports the role of SUMOylation as another mechanism to explain the differential properties of SERMs and SERDs. Beyond crosstalk with ubiquitination and degradation, SUMOylation can directly affect protein–protein interactions and protein–DNA interaction. Indeed, we observed that fulvestrant leads to rapid loss of binding to DNA after a transient recruitment phase, accompanied by chromatin compaction (44). Further characterization of the crosstalk between ERα SUMOylation and ubiquitination induced by AEs and of the relative contribution of each type of modification to transcriptional repression by SERDs will be important in order to better understand the mechanisms of action and tissue-specific effects of this class of AEs, whose use in the clinic is likely to increase sharply due to the recent development and promising clinical responses observed with new orally bioavailable molecules.

## Experimental procedures

### Reagents

E2, OHT, and Las were obtained from Sigma-Aldrich. Ra), Baz, and RU were obtained from Tocris. Fulvestrant (F) and GDC-0927 (GDC) were purchased from MedChemExpress, and AZD9496 (AZD) from Caiman Chemicals. All AEs were dissolved in DMSO to a concentration of 10^-2^ M and stored in aliquots in the dark at −20 °C until subsequent dilution in 95% EtOH to generate 10^-3^ M master mixes used for cell treatment at 1:1000 V/V dilution. Antibodies used in this study are described in Supporting Material (Table S1). The transfection reagent polyethylenimine (PEI) was ordered from Polysciences, Inc. Polyvinylidene difluoride membranes were purchased from EMD Millipore. Enhanced chemiluminescence (ECL) detection reagents were ordered from Bio-Rad.

### Cell culture

HEK293 (human embryonic kidney) cells were maintained in Dulbecco's modified Eagle's Medium (DMEM, Wisent) with phenol red supplemented with 10% FBS (fetal bovine serum, Sigma). MCF7 cells were maintained in Alpha Modification of Eagle’s Medium (Wisent) with phenol red supplemented with 10% FBS and with glutamine (2 mM, Wisent). All cells were maintained in a humidified 37 °C, 5% CO_2_ incubator. Cells were switched to DMEM without phenol red, supplemented with 10% of charcoal-dextran–treated FBS (FBS-T) and with glutamine (4 mM) 2 days before Western blot assays in MCF7 cells, BRET assays, and luciferase reporter experiments and 3 days before Western blot assays in HEK293 cells, then trypsinized, and seeded as specified below for each type of experiments.

### Western analysis

HEK293 cells were plated at 400,000 cells/well in 12-well plates in complete DMEM medium without red phenol. The next day, cells were transfected using pSG5-ERα or pSG5-ERβ (400 ng) with pcDNA3-Flag-SENP1 or the parental vector (800 ng) completed with salmon sperm DNA to 1.6 μg total using PEI [3 μg of PEI per μg of DNA (3:1)]. For the experiments with unfused chimeras (cloned in pSG5), cells were transfected with 2.6 μg total of DNA with PEI (3:1). Cells were treated with fulvestrant (1 μM) or vehicle (EtOH 0.095% with 0.01% DMSO final concentration) 2 days later for indicated times. For Western analysis of MCF7 cells, 500,000 cells/well in 6-well plates were seeded in complete DMEM medium without red phenol. Two days after the seeding, the cells were treated for 6 h with the SUMOylation inhibitor ML-792 followed by 1 h or 6 h of treatment with E2 (25 nM), the different AEs (1 μM), or vehicle (EtOH 0.095% with 0.01% DMSO at final concentrations). Cells were then collected in PBS 1× supplemented with N-ethyl-maleimide (20 mM final). Cell pellets were frozen at −20 °C until extracted using total lysis buffer (50 mM Tris-HCl pH 7.5, 150 mM NaCl, 5 mM EDTA, 2% SDS, 0.5% Triton, and 1% NP40) supplemented with aprotinin, leupeptine, and pepstatin (1 μg/ml final), N-ethyl-maleimide (20 mM final), and PMSF (1 μg/ml final). Pellets were resuspended in 50 to 100 μl of freshly prepared lysis buffer, depending on the size of the pellets, and sonicated with a Bioruptor (Diagenode) at 4 °C at medium potency for 15 min (30 s ON - 30 s OFF cycles). Samples were quantified using a Lowry assay (Bio-Rad). 15 μg of proteins in Laemmli buffer (1×) were loaded and separated by PAGE (8% acrylamide gels). After transfer onto polyvinylidene difluoride membranes, blots were blocked (5% milk in PBS 1×, Tween 0.05%) and incubated with primary and secondary antibodies diluted as described in Table S1 in blocking buffer. Immunodetection was performed using the enhanced chemiluminescence Clarity Western ECL substrate or Clarity Max Western ECL substrate (Bio-Rad) as recommended by the manufacturer, and imaging was performed using a ChemiDoc Imaging System (Bio-Rad).

### BRET assays

For BRET titration curves, HEK293 cells (1.25 10^6^ cells) were transfected in suspension in phenol red-free DMEM medium supplemented with FBS-T, with 50 ng of pcDNA3-ERα-RLucII or pcDNA3-ERβ-RLucII together with different amounts of pcDNA3-YFP-SUMO3 (0–800 ng in 2-fold dilutions) complemented to 1 μg with salmon sperm DNA. Transfections were performed using PEI (3:1), and cells were plated in 96-well white plates (Corning Incorporated). Two days after transfection, cells were switched to HBSS 1× supplemented with 4.5 g/L of dextrose and treated in quadruplicates with OHT, Ral, Las, Baz, AZD, RU, GDC or F (1 μM), E2 (25 nM), or vehicle (EtOH 0.095% with 0.01% DMSO) for 2 h.

For BRET kinetics or single point treatments, HEK293 cells (1.25 × 10^6^ cells) were transfected in suspension using PEI in DMEM medium without phenol red supplemented with FBS-T as above, with 50 to 400 ng of pcDNA3-ERα-RLucII, pcDNA3-ERβ-RlucII, or chimeras together with 500 ng of pcDNA3-YFP-SUMO1 or SUMO3, complemented with pcDNA3 empty vector and with salmon sperm DNA to 1 μg. Two days after transfection, cells were treated for different times with E2 (25 nM), AEs (1 μM), or vehicle (EtOH 0.095% with 0.01% DMSO) in quadruplicates for each time point (BRET kinetics) and at least in triplicates for single time point (2 h) assays in HBSS 1× supplemented with 4.5 g/L of dextrose before BRET measurements.

For testing the impact of wt or catalytically dead mutant PIAS proteins on ERα SUMOylation in the SUMO3 BRET assay, HEK293 cells (1.25 × 10^6^ cells) were transfected in suspension using PEI (3:1) in DMEM medium without phenol red supplemented with FBS-T, with a fixed amount of ERα (50 ng) and SUMO3 (500 ng) with or without overexpression (400 ng) of PIAS proteins (PIAS1, PIAS2α, PIAS2β, PIAS3, or PIAS4), catalytically dead mutants of PIAS proteins (PIAS1 C351S, PIAS2α or PIAS2β C362A, PIAS3 C308S or PIAS4 C342A) or empty vector (pCMV-Flag) complemented with salmon sperm DNA to 1.5 μg. Two days after transfection, cells were treated with fulvestrant (1 μM) or vehicle (EtOH 0.095% with 0.01% DMSO) in triplicates for 2 h in HBSS 1× supplemented with 4.5 g/L of dextrose before BRET measurements.

After treatment, total fluorescence was determined with a FlexStation microplate reader (Molecular Devices) using an excitation filter at 485 nm and an emission filter at 535 nm. Total luminescence was measured with a MITHRAS LB940 plate reader 5 min after the addition of coelenterazine H (5 μM final). BRET^1^ ratios were expressed as a function of the [acceptor]/[donor] expression ratios (YFP/Luc). Total fluorescence and luminescence measurements were used as a relative measure of expression of the acceptor and donor proteins, respectively.

### Luciferase assays

HEK293 cells (0.975 × 10^6^ cells) were transfected in suspension in phenol red-free DMEM supplemented with charcoal-stripped FBS (10%) and glutamine (4 mM) with the luciferase reporter vector p*GREB1*-ERE-Luc (1.25 μg), in which the strong proximal *GREB1* enhancer (−1.6 kb from the most upstream transcription start site, containing a perfect ERE sequence: GGTCAGAATGACCC) cloned upstream of a TATA box and of the luciferase gene, together with expression vectors for ERα, ERβ, chimeras or the parental pSG5 vector (0.3 or 0.75 μg), and a GFP internal control expression plasmid (pGFP-N2, 100 ng). Transfections were performed using PEI (3:1), and cells were plated in 96-well white plates (Corning Incorporated). Twenty-four hours after transfection, cells were treated in triplicate with E2 (2.5 nM), AEs (1 μM), or vehicle for 24 h. Cells were then harvested and assayed for luciferase activity. GFP signals were measured prior to luminescence assessment with a FlexStation microplate reader (Molecular Devices) at excitation/emission wavelengths of 355/538 nm. Cells were assayed for luciferase activity in the presence of a luciferin lysis solution (30 mM tricine, 1.61 mM (MgCO_3_)_4_•Mg(OH)_2_•5H_2_O, 4 mM MgSO_4_, 0.15 mM EDTA pH 7.0, 50 mM DTT, 0.81 mM coenzyme A, 0.7 mM D-luciferin, 0.8 mM ATP, 1% Brij58, 100 mM Trizma Acetate, 20 mM (CH_3_COO)_2_ Mg, and 2 mM EGTA in PBS 1×). Luminescence measurements were acquired after 10 min incubation at room temperature on a MITHRAS LB940 multidetector plate reader (Berthold Technology) in the absence of an emission filter. Relative luciferase signals (Rel Luminescence) were obtained by normalization to GFP expression.

### Statistical analyses

Curve fitting and statistical analyses were performed in GraphPad Prism 6.07. All measurements were performed in three to eight technical replicates and repeated two to four times (different transfections). For statistics on the impact of ligands on receptor/chimera activity (BRET or LUC assays), significance was determined using a Holm–Sidak’s multiple *t* test assuming the same scatter (SD). For testing the impact of each AE *versus* no treatment on ERα and SUMO3 interaction in BRET assays, a one-way ANOVA followed by Dunnett’s multiple comparison test was used. For titration experiments, Bmax values computed from nonlinear regression were analyzed by one-way ANOVA followed by Tukey’s multiple comparison test. Finally, for testing the impact of PIAS proteins and catalytically dead mutants on ERα SUMOylation by SUMO3 in BRET assays, a two-way ANOVA followed by a Tukey’s multiple comparison test was used. Adjusted *p*-values lower than 0.05 were considered significant.

## Data availability

All data are contained within the manuscript.

## Supporting information

This article contains supporting information.

## Conflict of interest

The authors declare they have no conflict of interest with the contents of this article.
