# Supplementary material for: The AF-2 cofactor binding region is key for the selective SUMOylation of estrogen receptor alpha by antiestrogens
Source: J Biol Chem. 2022 Nov 30;299(1):102757. doi: 10.1016/j.jbc.2022.102757 (PMC9823126; doi:10.1016/j.jbc.2022.102757)
Supplement: Supporting information [file mmc1.pdf]

The AF-2 cofactor binding region is key for the selective SUMOylation of estrogen receptor alpha by antiestrogens

Amandine Vallet[1*], Mohamed El Ezzy[1*], Marine Diennet[1], Salwa Haidar[1], Michel Bouvier[1,2], Sylvie Mader[1,2,**]

SUPPORTING INFORMATION

Experimental Procedures

See *Experimental Procedures* in main text.

Figs. S1, S2, S3 and S4

Table S1

Fig. S1: Comparison of Bmax values from titration curves for ERα/β SUMO BRET assays in the presence of fulvestrant or other antiestrogens.
(**A**) Comparison of Bmax values obtained after a non-linear regression analysis in GraphPad Prism 6.07 of the BRET SUMO3 titration curves with ERα/ERβ donor constructs after treatment with vehicle (0) or fulvestrant (F) (Figure 1E). The graphs are representative of 3 independent experiments performed in quadruplicates (mean values ± SEM). (**B**) Comparison of Bmax values obtained after a non-linear regression analysis in GraphPad Prism 6.07 of the BRET SUMO3 titration curves of ERα after treatment with different antiestrogens (Figure 2E). Statistical analyses were performed using one-way ANOVA and Tukey's multiple comparison test in GraphPad Prism 6.07 (*: $p<0.05$).

Fig. S2: The ERα identity of the EF domain in chimeras is sufficient for induced SUMOylation by fulvestrant. (A) BRET titration curves were performed by transient transfection into HEK293 cells of a fixed amount of ERα-RLucII, ERβ-RLucII or chimeras and varying amounts of YFP-SUMO1, either in the presence of fulvestrant (1 μM) or vehicle for 2 h. The x axis represents the measured YFP over RLucII expression and the y axis the net BRET ratios measured in live cells after addition of CoelH. The curves are a compilation of 3 independent experiments, each point representing the mean values of technical quadruplicates. Bmax values (showed in graph **B**) derived from non-linear regression analysis were calculated for each biological replicate and statistical analysis was performed using one-way ANOVA and Tukey's multiple comparison test in GraphPad Prism 6.07 (*: $p<0,05$).

Fig. S3: BRET of ERα with SUMO1 is dependent on covalent interaction with SUMO and induced by PIAS1 overexpression. ERα fused C-terminally (ERα-RLuc) or N-terminally (RLucERα) with RLucII was co-transfected with SUMO1 or the SUMO1G mutant that cannot be covalently bound to substrates, in the absence or presence of PIAS1 in HEK293 cells. After treatment with fulvestrant (1 μM) or vehicle for 2 h, net BRET ratios were assessed in live cells. The graphs are representative of 2 independent experiments performed in triplicates (mean values ± SEM). Statistical analyses were performed using Holm-Sidak's multiple t test assuming the same scatter in GraphPad Prism 6.07 (*: $p<0,05$).

Fig. S4: The ERα identity of helices H3-H4 in the ligand binding domain is necessary and sufficient for modification of chimeras by SUMO1 in the presence of fulvestrant. (A-D) SUMO1 BRET assay with ERα/β chimeras in HEK293 cells treated or not with fulvestrant (2h, 1 μM). The graphs are representative of 3 independent experiments performed in triplicates (mean values ± SEM). Statistical analyses were performed using Holm-Sidak's multiple t test assuming the same scatter in GraphPad Prism 6.07 (*: $p<0,05$).

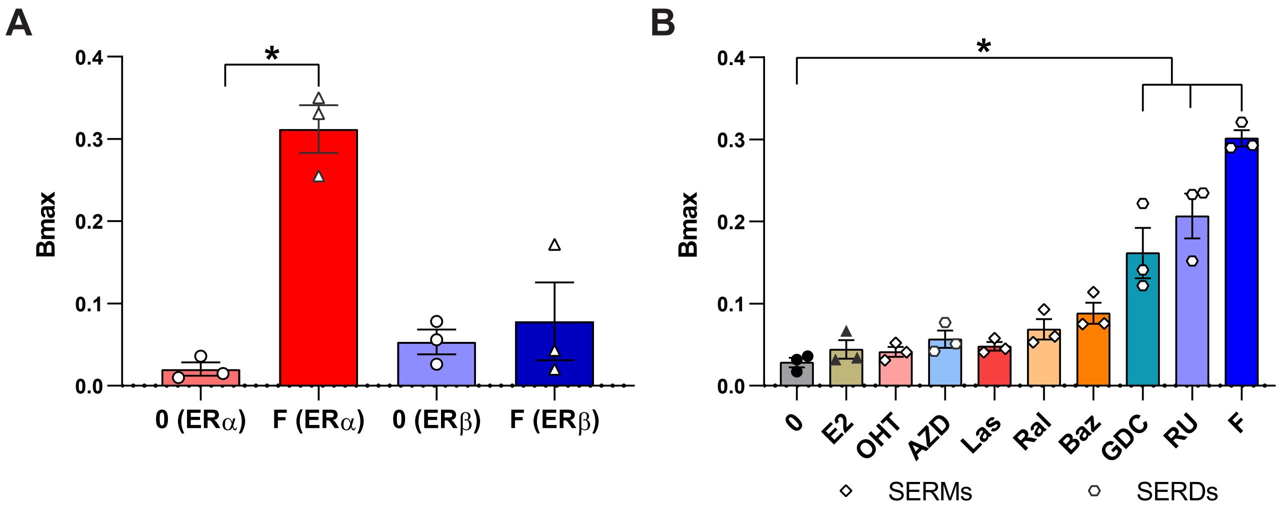

A
B
*
Bmax
0.4
0.3
0.2
0.1
0.0
0 (ERα)
F (ERα)
0 (ERβ)
F (ERβ)
0
E2
OHT
AZD
Las
Ral
Baz
GDC
RU
F
SERMs
SERDs

Figure S1

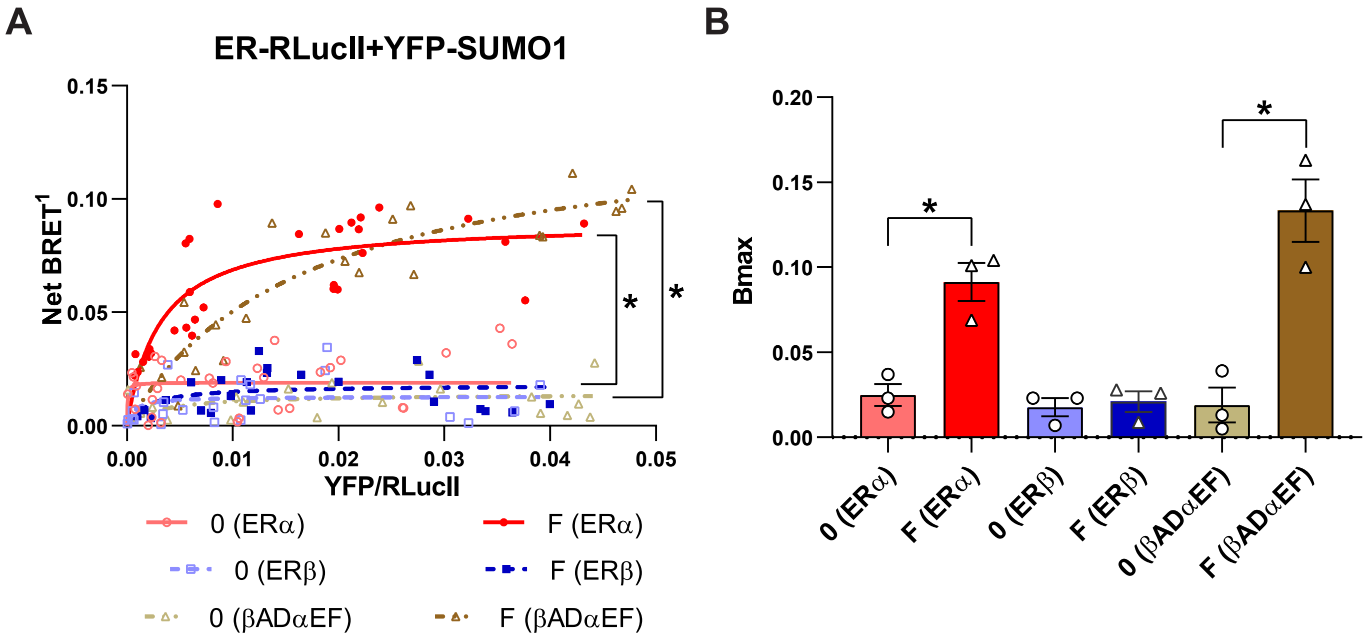
A
ER-RLucII+YFP-SUMO1
Net BRET-1
0.15
0.10
0.05
0.00
0.00
0.01
0.02
0.03
0.04
0.05
YFP/RLucII
*
*
0 (ERα)
F (ERα)
0 (ERβ)
F (ERβ)
0 (βADαEF)
F (βADαEF)
B
Bmax
0.20
0.15
0.10
0.05
0.00
*
*
0 (ERα)
F (ERα)
0 (ERβ)
F (ERβ)
0 (βADαEF)
F (βADαEF)

Figure S2

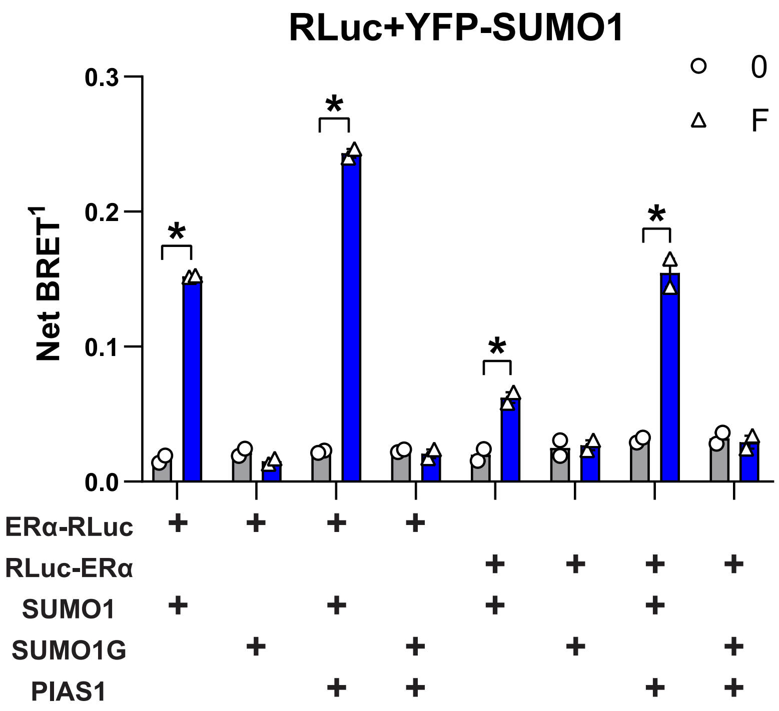
RLuc+YFP-SUMO1
0
F
0.3
0.2
0.1
0.0
Net BRET-1
*
*
*
*
ERα-RLuc
RLuc-ERα
SUMO1
SUMO1G
PIAS1

Figure S3

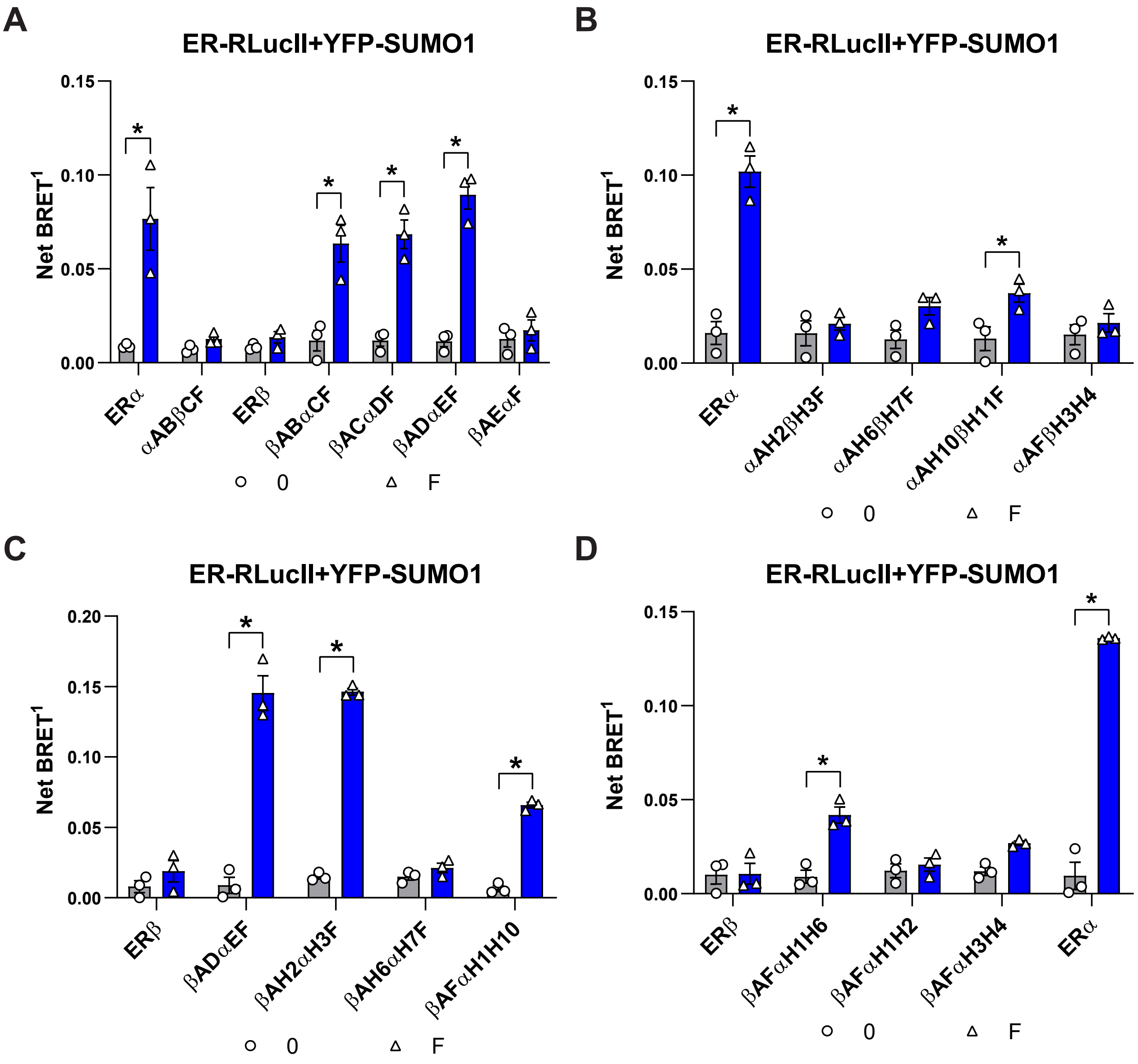
A
ER-RLucII+YFP-SUMO1
Net BRET1
0.15
0.10
0.05
0.00
ERα
αABβCF
ERβ
βABαCF
βACαDF
βADαEF
βAEαF
0
F
B
ER-RLucII+YFP-SUMO1
ERα
αAH2βH3F
αAH6βH7F
αAH10βH11F
αAFβH3H4
C
ER-RLucII+YFP-SUMO1
0.20
ERβ
βADαEF
βAH2αH3F
βAH6αH7F
βAFαH1H10
D
ER-RLucII+YFP-SUMO1
βAFαH1H6
βAFαH1H2
βAFαH3H4

Figure S4

Target	Provider	Clone	Species	Epitope	Dilution
**ERα**	Millipore 04-820	60C	Rabbit monoclonal	Raised agaisnt a peptide corresponding to amino acids 13-32 (C-ALLHQIQGNELEPLNRPQLK) of human ERα.	1:2000
**ERβ**	Invitrogen MA5-24807	PPZ0506	Mouse monoclonal IgG_{2b}	Raised against a peptide in the N-terminus (aa 2-88) of hERβ.	1:1000
**SENP1**	Santa Cruz sc-271360	C-12	Mouse monoclonal IgG1	Raised against amino acids 361-425 of human SENP1.	1:1000
**β-Actin**	Santa Cruz sc-373853	AC-15	Mouse monoclonal IgG1[illegible]	Raised against a slightly modified synthetic peptide corresponding to cytoplasmic β-actin.	1:10000

Table S1: Antibodies used in this study.
